# Vertical lossless genomic data compression tools for assembled genomes: A systematic literature review

**DOI:** 10.1371/journal.pone.0232942

**Published:** 2020-05-26

**Authors:** Kelvin V. Kredens, Juliano V. Martins, Osmar B. Dordal, Mauri Ferrandin, Roberto H. Herai, Edson E. Scalabrin, Bráulio C. Ávila

**Affiliations:** 1 Graduate Program in Informatics (PPGia), Pontifícia Universidade Católica do Paraná, Curitiba, Paraná, Brazil; 2 Polytechnic School, Centro Universitário UniDomBosco, Curitiba, Paraná, Brazil; 3 Department of Control, Automation and Computing Engineering, Universidade Federal de Santa Catarina (UFSC), Blumenau, Brazil; 4 Graduate Program in Health Sciences, School of Medicine, Pontifícia Universidade Católica do Paraná (PUCPR), Curitiba, Paraná, Brazil; King Abdulaziz University, SAUDI ARABIA

## Abstract

The recent decrease in cost and time to sequence and assemble of complete genomes created an increased demand for data storage. As a consequence, several strategies for assembled biological data compression were created. Vertical compression tools implement strategies that take advantage of the high level of similarity between multiple assembled genomic sequences for better compression results. However, current reviews on vertical compression do not compare the execution flow of each tool, which is constituted by phases of preprocessing, transformation, and data encoding. We performed a systematic literature review to identify and compare existing tools for vertical compression of assembled genomic sequences. The review was centered on PubMed and Scopus, in which 45726 distinct papers were considered. Next, 32 papers were selected according to the following criteria: to present a lossless vertical compression tool; to use the information contained in other sequences for the compression; to be able to manipulate genomic sequences in FASTA format; and no need prior knowledge. Although we extracted performance compression results, they were not compared as the tools did not use a standardized evaluation protocol. Thus, we conclude that there’s a lack of definition of an evaluation protocol that must be applied by each tool.

## Introduction

*Next-Generation Sequencing* (NGS) [1, 2] made possible a significant advance in the genetic sequencing of thousands of organism genomes. Besides, the sequencing capacity has doubled every seven months [3] in a way that, in the next 20 years, the global sequencing capacity might reach 1 billion people annually [3]. Therefore, data storage costs could be a bottleneck when compared to sequencing prices [4]. Thus, rather than merely increasing space [5, 6], more efficient data storage methods are needed [7, 8]. Given such demand, data compression approaches are considered a very efficient alternative.

Data compression could be applied to various biological data types represented by different file formats, such as FASTQ for sequenced reads, BAM/SAM for aligned data, VCF for haplotype or sequence variation. Among these file formats, compression tools for NGS data in FASTQ or data alignment in BAM and SAM files were recently benchmarked by the MPEG HTS [9] group. VCF files [10] or graph-based structures are commonly used to represent genetic variations between different sequences (including haplotypic variation). For haplotypic variations, sequenced reads are mapped to a reference genome (reference-based), or are used for a de novo assemble to create a consensus sequence that is then mapped to a reference sequence [11]. The most common use of VCF files are for haplotypic studies in *Genome-Wide Association Studies* (GWAS), such as the 1000 Genomes Project [12], UK10K [13], GoNL [14] or HRC [15]. Haplotype variation data, stored in VCF files can be compressed by some tool as GTC [16], BGT [17], SeqArray [18], and PBWT [19] or, when stored in a graph-based format by tools gPBWT [20] and BFT [21].

Another type of file format is FASTA, commonly used to represent a partially or fully assembled sequence from an organism’s genome [22]. A genome sequence could be obtained by resequencing or by a de novo-based assembly. In the resequencing process, sequenced reads are mapped to the corresponding organism reference genome. In the de novo-based assembly process, the sequence reconstruction is an overlapping-based sequenced reading, creating contigs and scaffolds [23].

A de novo-based assembled genome, compared to a reference-based, turns possible to identify complex structural variations or large repetitive regions [23] to create a complete map of genetic variations [24]. For this reason, de novo-based approaches are being widely used for the correct identification of large sequence variations. Considering the importance of de novo-based assembled sequences, normally stored in FASTA format, several compression tools were proposed. As defined by the authors of Biocompress [25], the first genomics sequence compression tool, the compression process could be performed by two modes: horizontal and vertical. In horizontal mode, each genomic sequence is compressed using its self-contained information as a search space [25]. In vertical mode, also known as delta or differential compression, the content of one or more sequences can be used as a search space to detect shared repetitive segments. Although for some cases, the used nomenclature for genomic data compression considers vertical mode equivalent to referential mode, the second corresponds to a subtype of the first [26, 27]. Vertical compression tools are designed for genomes of same species, as they have high genomic similarity, such as between humans that share 99,5% of all their genetic material [28]. Thus, it would be possible to store only the 0.5% differences [27].

Previous reviews had been published (Table 1) but they covered different topics such as important bioinformatics areas in which compression techniques could be applied [26, 29, 30] or NGS data compression [31]. Furthermore, some reviews [4, 27, 32–34] covering the topic vertical genomic data compression consider a small number of tools to be compared, less than 10.

**Table 1:** Genomic data compression reviews.

Publication Year	Title	Published Journal
2009	Textual data compression in computational biology: a synopsis	Bioinformatics
2009	Data Compression Concepts and Algorithms and Their Applications to Bioinformatics	Entropy
2012	Recent Directions in Compressing Next Generation Sequencing Data	Current Bioinformatics
2012	Textual data compression in computational biology: Algorithmic techniques	Elsevier Computer Science Review
2013	Data compression for sequencing data	Algorithms for Molecular Biology
2013	Compressive biological sequence analysis and archival in the era of high-throughput sequencing technologies	Briefings in Bioinformatics
2013	DNA Lossless Compression Algorithms: Review	American Journal of Bioinformatics Research
2013	High-throughput DNA sequence data compression	Briefings in Bioinformatics
2014	Trends in Genome Compression	Current Bioinformatics
2016	A Survey on Data Compression Methods for Biological Sequences	Information

Due to the importance of compression methods and the high variability of existing methodologies, we performed a *Systematic Literature Review* (SLR) to identify existing compression tools for the compression of a collection of assembled genomic sequences.

Thus, to create the macro vision of the main characteristics of the computational compression tools, the following research questions were enunciated:

**RQ1**: Which are the existent tools for *Vertical Genomic Data Compression* (VGDC), and in which way do they process the sequence content and how they provide access to compressed data?
**RQ2**: Which are the techniques used by VGDC tools to detect shared segments and to encode the final result?

In our review, we performed an in-depth review of 32 vertical compression tools. Of these, 16 (50%) were published in the last 3 years. We list the existing tools for vertical genomic data compression. Next, we describe each tool process the sequence content and how they provide access to the compressed data. Then, we describe the used techniques by the tools to detect shared segments and to encode the final result.

## Results

### Article selection and overview

**Fig 1:**
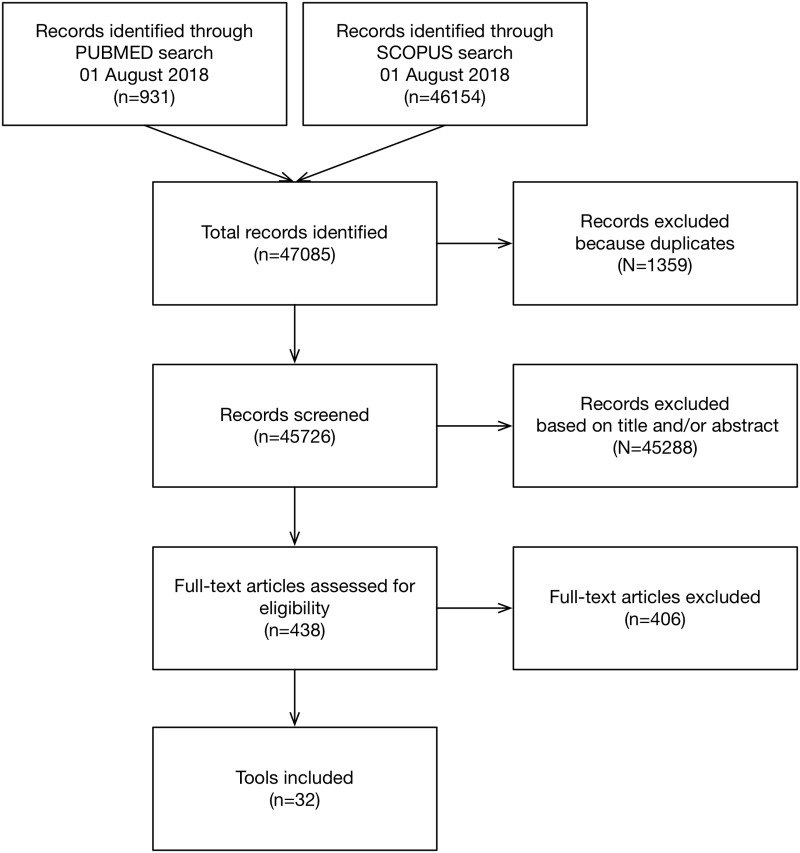
Flowchart of study identification and selection process.

This systematic literature review was conducted following the protocol defined in the previous section. The search was performed on August 1, 2018, and after the removal of duplicated papers, 45726 unique papers were obtained. After applying the inclusion criteria, 32 tools were filtered to be assessed by this review (Fig 1). Quantitative filters (impact factor, the number of citations or journals in which the papers were published) were not applied as exclusion criteria in this review. From this point, each research question was answered by analyzing the full content of the selected papers.

**RQ1**: **Which are the existent tools for *Vertical Genomic Data Compression* (VGDC), and in which way do they process the sequence content and how they provide access to compressed data?**

**Fig 2:**
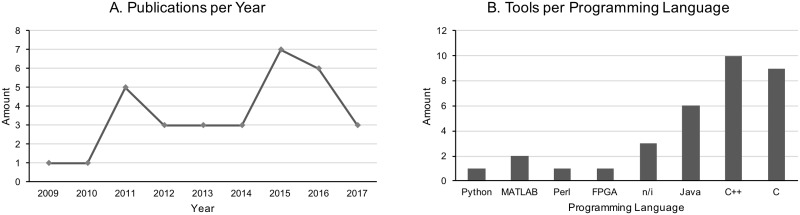
Tools for VGDC. Considering (A) Amount of Publications per Year and (B) Amount of Tools per Programming Language. The total number of tools is greater than 32 because the HiRGC tool has versions in C ++ and Java.

From the selected papers, 32 genomic data compression tools were identified (Table 5), and the following information was extracted: publication date, programming language, the highest and lowest compression ratios (ratio between the final size and initial size of the compressed genomic sequence), compression scheme, supported alphabet, compression of sequence header, external memory usage, and available method for compressed data access (Table 5). Although the human genome was concluded in 2001 [35, 36], all the vertical genomic compression tools were reported only after 2009 (Fig 2A).

We also observed that about 57, 5% of tools were written using the programming languages C or C++ (Fig 2B). Although the authors did not justify the reason of using a specific programming language, both mostly used languages are based on architecture-based compiled codes that, in a general manner, allows faster execution times when compared to interpreted codes [37]. Another extracted information was the compression ratio [see Additional file 1: Table S2] that is defined as the ratio between the uncompressed size and compressed size. Such information was extracted directly from the data provided by each paper. We prioritized the extraction of compression results of H. Sapiens genome sequences. However, the tools RCC, RLZAP, Kanika, et al., RLZ 1.4, RLZ RePair and Project DNA Compression did not present any result using the H. Sapiens genome as a reference dataset [Additional file 1: Table S2, highlighted in bold].

However, the data is not useful for direct comparison between tools since they were generated at different conditions. A wide variation of compression ratios among the tools might be noticed. This is due to the lack of dataset homogenization for assessing compression ratio. This same observation is supported by other authors [27, 33], that also observed the lack of a standardized protocol for performance evaluation. Taking altogether, the following differences were observed: a) the use of different sequences, from large genomes of animal and plants to small genomes of microorganisms, b) the use of distinct methods for gathering and/or presenting compression performance evaluation metrics, and c) the use of a limited number of specialized compression tools and, for some cases, using generic tools like GZIP, making it difficult to perform a comparative and conclusive performance evaluation.

As a systematic literature review, it was not the aim of this review to perform a performance evaluation as performed by some others genomic data compression surveys [9, 22]. The first [9] one focus only in *High-throughput sequencing* (HTS) data compression tools and in the second [22] there is an attempt to compare different vertical compression tools, but it was restricted to only two tools, the GRS [38], and the GReEn [39]. Additionally, the used test dataset, although having sequences varying the species and genome size, did provide detailed information about the sequence characteristics, such as their similarity degrees and types of variations, if *single-nucleotide polymorphism* (SNP), *inserts and deletions* (INDEL) or *structural variations* (SV).

Based on these previous reports, it becomes crucial to define a standard protocol to ensure conclusive and comparable performance evaluation [40]. Therefore, some of the following definitions must be standardized: a) method for a tool execution; b) metrics (such as compression time, memory requirements and disk usage) to be collected; c) time points for metrics collection; d) information that must be reported in order to guarantee reproducibility of the experiments; e) standardizing the results presentation; f) datasets that must be used in the evaluation. When analyzed the compression ratio described by each assessed tool, we found a an accretion on the final size of the compressed input dataset in hundreds of times, and in some cases even thousands of times. As reported by the authors, the power of compression is due to the high level of similarity between sequences. However, these tools did not perform an in-depth analysis, correlating the characteristics of the sequence and their similarity levels with the efficiency of compression ratios, for better or worst results. The definition of a set of parameters corresponding to the dataset characteristics could improve the compression ratio and also predict which could be the better approach to be used for.

For example, in the results of the ABRC tool, it was observed a very different compression ratio when comparing compression results of same species genomes. For a dataset of H. Sapiens sequences, the tool reached a compression ratio of 397:1 and, by after compressing *Saccharomyces Cerevisiae* sequences, such ratio reduced to 61:1. Based on these reported results, we raised the following questions: Which characteristics between these two datasets are causing such difference in compression ratio? Would it be only the quantity of repetitions or any other type of repetitive region? Alternatively, would that be a result of other types of genetic variation, such as those resulting from SNP, INDEL or SV?

According to our analysis, the compression tools did not follow a standard for the representation of the input dataset, the method to present the compression results, and the used metrics/approach for compression evaluation. It was also observed that many tools used the human genome KOREF_20090224 [41] for their performance tests, but each tool reported a different genome size (Table 2). Although we could list several reasons for that difference, such the use of a different file system to store the files or a preprocessing step before the data compression, it shows the need for a dataset representation standard.

**Table 2:** Informed size for KOREF_20090224 human genome.

Tool	Size
HiRGC	2.987 MB
FM-context	3.080.419.480 bytes
MLF	3.080.436.051 bytes
NRGC	2.938 MB
SLF	3.080.436.051 bytes
ERGC	2.938 MB
iDoComp	3.100 MB
DnaCompact	2.937,7 MB
Dai et al.	3.080.436.051 bytes [t]
COOL	2.938 MB [b]
GRS	2.986,8 MB
GReEn	3.080.436.051 bytes
GDC-0.3	3.131,78 MB
Actual size^a^	3.131.776.827 bytes

**Tool**: Name of tool or quotation of it when there is no name; **Size**: Size of KOREF_20090224 genome, as it is presented by each tool in its original papers.

Notes:

^a^—Total value, in bytes, by adding the total bytes to the FASTA files available in ftp://ftp.kobic.kr/pub/KOBIC-KoreanGenome/KOREF_20090224/fasta/ at 26/09/2016.

**Fig 3:**
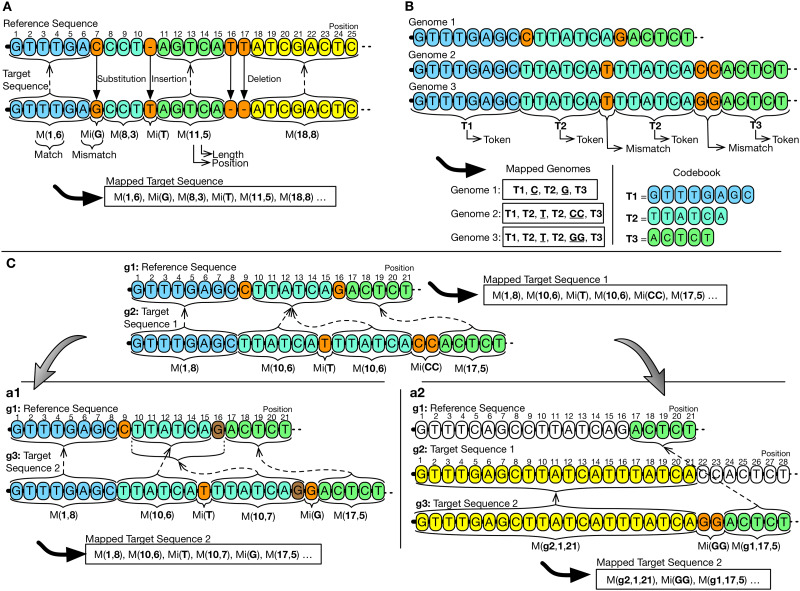
Compression schemes. (A) Referential pair-based. The target genome sequence will be compressed using information extracted from the reference genome sequence. The goal of this process is to find shared segments. The goal of this process is to find shared segments. The final result is a map (Mapped Target Sequence) in which matches replace such shared segments, and the not shared are written in raw, as a mismatch. In this example, the match is composed of two integers, the first being the position where the shared segment begins in reference and the second representing the size of the match. (B) Dictionary collection-based. In this example, three genomes are compressed at the same time. In the first moment, the process searches for shared segments and stores them in a structure called codebook. Then, to obtain the mapped genomes, it rewrites sequences, replacing segments by indexes of tokens previously created. Token indexes and mismatches symbols will compose the final mapping. In this example, we consider that to be converted as a token, a shared segment must be larger than three bases. (C) Referential collection-based single/multi-reference. In this example, sequences g1, g2, and g3 are compressed. On the first step (C), g2: Target Sequence 1 is compressed concerning g1: Reference Sequence and generates a mapping between them. The second step represents behavior or single-reference (c1) or multi-reference (c2). In the case of single-reference (c1), the process is similar to the previous step (C), in which sequence g3: Target Sequence 2 is compressed based on the same g1: Reference Sequence used in (C). In multi-reference (c2), however, the compression process will try to find shared segments using both g1: Reference Sequence and the ones already compressed, in this case, g2: Target Sequence 1. Thus, matches can point to any other sequence of the collection.

Besides the differences observed in the described pipelines for data compression, we also identify and described which the tools currently use compression schemes. A detailed analysis of the papers led to the identification of 3 different schemes as follow (Table 5):

- Referential: Given a collection of genomic sequences, the referential compression, also known as relative, delta or differential [27], consists on the selection—automatic or manual—of a genomic sequence as reference. The remaining sequences, called targets, are then compared to the reference to detect and store only the different parts [22]. These stored parts are replaced by identifiers, called matches, within target sequences, which points to segments of the reference sequence [33]. Fig 3A illustrates an example of referential compression, in which the first match, m(1,6), indicates that the shared segment *GTTTGA* is initiated in the reference sequence, position 1, having 6 nucleotides. Compression is by achieved by using the information shared between the reference sequence and the corresponding target sequences [32]. Such scheme is implemented by the following tools: RCC, HiRGC, FM-context, MLF, RLZAP, NRGC, RCSCS, DNAComp, SLF, JDNA, Arram et al., ERGC, GDC 2.0, CoGI, iDoComp, DNAC-K, RLZ 1.4, FRESCO, DnaCompact, Dai et al., ABRC, COOL, GRS, GDC 0.3, RLZ-Opt, RLZ-RePair, RLZ and Project DNA Compression;
- Dictionary: The basic idea of this scheme is to find repeated recurrent data portions (i.e., substrings), remove them from the main content and store them as indexed tokens in a dictionary, called codebook. This pieces of recurring data are replaced by its corresponding stored indexes from the codebook [42]. The compression will become effective when less space is required to store the indexes compared to the space for the tokens [43]. Fig 3B illustrates how a dictionary-based compression scheme works. In this scheme, the tool passes linearly through the three genomic sequences to detect shared segments between them. Detected segments are then moved in, as tokens, to the codebook. Here, the token T1 is composed by the bases *GTTTGAGC* and is found within all three sequences. The tools that implement the dictionary-based concept are COMRAD and Kanika et al [44]. Although Kanika and colleagues had described their tool as a referential-based schema, there is an initial step to create a reference set. Such reference is composed of shared blocks that are extracted from a set of random-defined reference sequences. These blocks, corresponding to DNA parts, are then localized in target sequences to be replaced by corresponding fingerprints. In this dictionary-based schema, the reference set is used as a codebook, and the tokens and fingerprints correspond to indexes;
- Statistical: instead of implementing a data transformation phase, the statistical-based schema directly applies the coding over the input genomic sequences. This scheme uses the information on the probability for a given nucleotide to occur within the sequence. Then, it can determine a unique code-word, which may be of variable size (proportional to the size of the alphabet). This code-word will be part of the final compressed file [45]. The tool GReEn [39] is based on an arithmetic coding model (detailed in [46]), that uses the reference sequence to create a dynamic probabilistic model. The model is used to compress the target sequences by generating a numeric interval to reconstruct the original sequence. The GeCo tool [47, 48] also uses an arithmetic coding schema. Its content corresponds to the occurrence probability of each nucleotide happen, that is calculated by two models, one based on *Finite-Context Models* (FCMs) and a second based on an *Extended Finite-Context Models* (XFCMs). For GReEn and GeCo tools, models are created and updated using information extracted from the reference sequence. Therefore, the better the capacity of the model to predicting the next nucleotide, the lesser the quantity of data to be stored [49].

As a vertical-based compression scheme, the tools require the availability of the reference sequence for the decompression process or the dictionary in the case of dictionary-based tools. We also investigated the number of genomic sequences involved in the compression process, that can be based on two different methods:

- Pair-based: compression method that uses as input data a single target genomic sequence to-be-compressed [50]. When a set of targets sequences are available, each individual sequence is separately compressed [50] using a reference sequence that must be available for the other targets sequences to-be-compressed or for decompression [25, 51] (Fig 3A);
- Collection-based: compression method that uses as input data a collection of genomic sequences to-be-compressed. The collection is analyzed to find for shared segments with only one reference sequence [52]—single-reference, or with a collection of them—multi-reference [29] (Fig 3C). Depending on the number of involved sequences, different compression steps may be applied. On the first step (Fig 3C) both approaches, single and multi-reference, perform the same search procedures through shared segments when there are only one reference sequence and one target sequence (Target Sequence 1). In the second step, the search procedure for shared segments becomes different. For a single-reference compression, a second target sequence (Fig 3c1, Target Sequence 2) is compressed using the same reference sequence used by Target Sequence 1. For the multi-reference compression (Fig 3c2), the Target Sequence 2 uses the information contained both in Reference Sequence and in Target Sequence 1. Target Sequence 1 is also incorporated within the set of compressed sequences and used as a source of information (search space). Due to the existence of more sequences that can be used as a reference, the probability of finding more shared segments increase.

Among the assessed tools (Table 5), only 6, GDC 2.0, DNAComp, DNAC-K, FRESCO, COMRAD e GDC 0.3, implement a collection-based compression method using the multi-reference approach. Preliminary results suggest that these 6 methods are promising as they have shown better results when compared to other approaches [50, 53]. A particular case is the RCC [54] tool, that groups the target sequences into clusters and, for each cluster, generates one artificial reference sequence (consensus sequence). Next, each target sequence is compressed using its corresponding reference sequence. Then, even though there is more than one reference sequence, each cluster makes use of only a single reference at a time, so we consider it to be a single-reference approach.

Another critical point in compressing genomic data is the form in which they process and provide access to a compressed FASTA file representation. This representation has 2 information for each sequence. The first information is a header line, corresponding to the sequence description/identification, beginning with the symbol “>”, and the second information corresponds to the sequence content in nucleotides. The FASTA representation is not case-sensitive, although some tools also handle this information. In a FASTA file having a genomic sequence, the header has an average size smaller than 256 symbols. Thus, it is tiny compared to the DNA sequence content it represents but required for recovering the compressed sequences. Therefore, the sequence header can be stored separately, without applying a proper compression schema.

Although essential for compressed data recovery, only a few tools store header information, which are: HiRGC, NRGC, JDNA, ERGC, GDC 2.0, iDoComp, DNA_COMPACT, COOL, GDC 0.3 and GRS. Other investigated tools either discard the header (MLF, Arram et al., GeCo, DNAComp, SLF, CoGI, ABRC, GReEn and Project DNA Compression), or do not mention how they treat the sequence header (RCC, FM-context, DNAC-K, RLZ 1.4, FRESCO, Dai et al., COMRAD, RLZ-opt, RLZ-RePair, and RLZ). The heterogeneity found in the way the tools process the sequence header makes it challenging to assess the performance of a specific tool precisely. For example, in the tests described by iDoComp tool, the authors ignored the size of the sequence header for small datasets to be able to make results comparable with other tools.

In addition to the sequence header, another essential characteristic for the compression of genetic data is the alphabet, used to represent the DNA sequence in a FASTA file. Four symbols compose the smallest required alphabet to represent a DNA sequence: *A*, *T*, *C* and *G*. Among the 32 assessed tools, 15 of them (HiRGC, FM-context, MLF, NRGC [55], SLF, ERGC, GDC 2.0, iDoComp, DnaCompact, Dai et al., ABRC, COOL, GRS, GReEn e GDC 0.3) do not impose any kind of alphabet restriction, being therefore capable of considering any symbol of ASCII table and also being case-sensitive. The only exception occurs for the tools COOL and ABRC. COOL converts all symbols to uppercase and ABRC tool did not specify whether it uses this information. Among the other tools, two tools accept IUPAC alphabet (COMRAD and DNAC-K), and other 9 tools (RLZAP, GeCo, JDNA, CoGI, RLZ 1.4, FRESCO, RLZ-opt, RLZ-RePair e RLZ) that consider an alphabet of reduced size and composed by the symbols *ATCGN*. The tools RCC, Arram et al., GeCo, DNAComp e Project DNA Compression are restricted to only 4 symbols, corresponding to the *ATCG* nucleotides.

Besides the analysis of the treatment of FASTA content, we also investigated the computational demands of each compression tool, such as the use of secondary memory (disc). When used in an appropriated way, secondary memory provides the ability for a compression tool to be able to manipulate more massive datasets without restricting its use to the main memory (RAM) size. A well projected and implemented secondary memory use should not jeopardize the main memory of the system since its disorganized use could force the compression tool to use the virtual memory (swap) from the Operating System, and then delaying the compression process. Therefore, when compressing big datasets in limited computational environments, a compression tool should be able to self-manage the use of computational resources. Among the assessed tools, this functionality was implemented by GeCo, NRGC, JDNA, ERGC, ABRC, and COMRAD. In GeCo, a new proposal was implemented using hash tables, called cache-hash, which are not required to be stored entirely in the computer’s main memory. Instead, the tool keeps stored within main memory only the most recent entries. In the tools NRGC, JDNA, and ERGC it is implemented an on-demand loading strategy of the to-be-compressed genomic sequences. In this method, the compression scheme works by keeping the remaining of the to-be-compressed data in the secondary storage. The ABRC tool implements a slightly different approach by loading into main memory only the index parts necessary for mapping. The COMRAD tool uses a similar approach, however during compression it processes each genomic sequence independently of the others. This strategy reduces the total amount of main memory required for compression.

Once the genomic data has been compressed, we also analyzed how the tools extract part or entire compressed sequences. This may be either the extraction of an entire sequence from a collection of compressed sequences or the extraction of a segment from a compressed sequence. Such extraction may require the decompression of the entire collection of compressed sequences (Total Recovery) or allow only the desired sequence to be decompressed (Single Recovery), the latter also is characterized by non-sequential data access [27]. Among the assessed collection-based tools, the single recovery functionality is available within the tools GDC 2.0, COMRAD, RLZAP, RLZ 1.4, GDC 0.3 and RLZ.

In the GDC 2.0 tool, single recovery is possible if only one reference sequence is used, otherwise, more than one sequence should be decompressed. The COMRAD tool also implements a single recovery process to extract the sequence of interest but requires that the full contents of the codebook and compressed sequences be stored in main memory. In this method, only the necessary tokens from the sequence to-be-decompressed will have their corresponding nucleotides extracted. The other tools, RLZAP, RLZ 1.4, GDC 0.3 and RLZ, implement, on single recovery form, both partial and complete sequence extraction. Data structures based on self-indexes are the broad approach used for Single Recovery [56–58].

**RQ2**: **Which are the techniques used by VGDC tools to detect shared segments and to encode the final result?**

**Fig 4:**
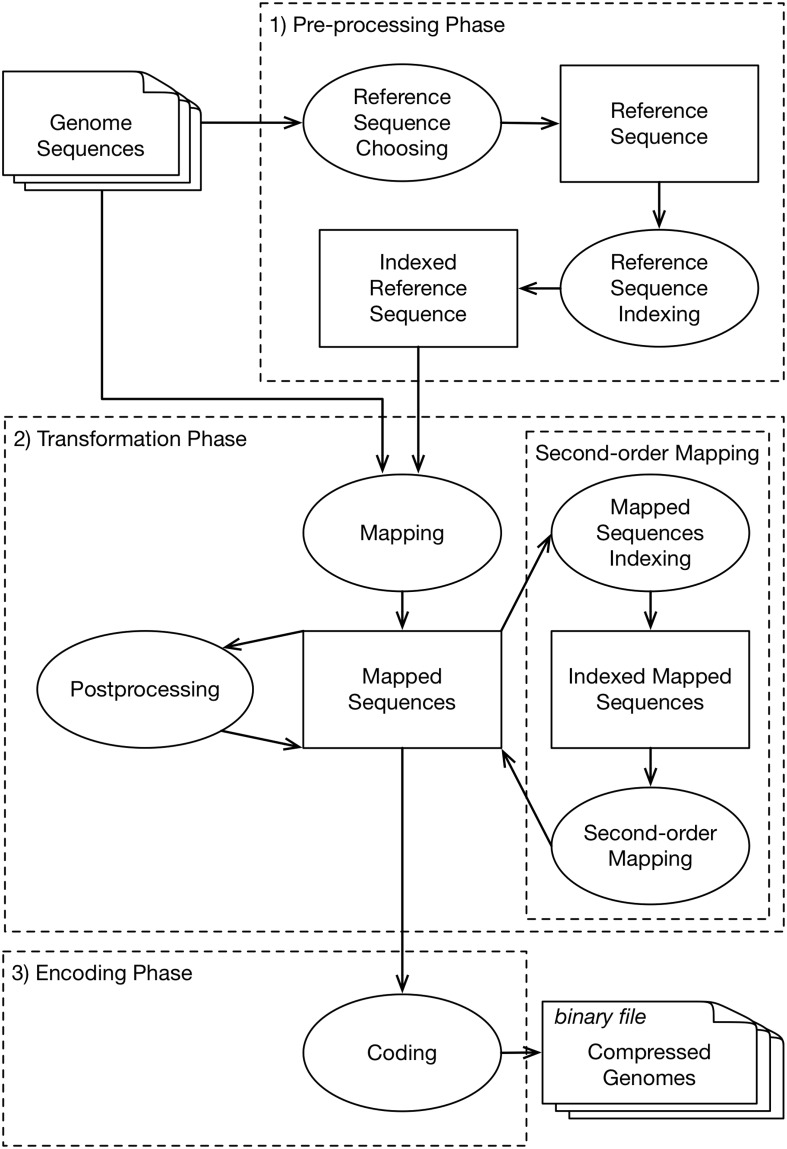
Conceptual compression flow. The conceptual vision of the activities that can be executed by a VCGD tool to compress DNA sequences. 1) Preprocessing Phase, initial phase to select the sequence that will be used as reference and index it. Some tools do not have an automatic process to select references. In these cases, tools expect the reference to be manually informed. 2) Transformation Phase, phase in which tools explore characteristics inherent to DNA sequences that, due to being all from the same or correlated species, tend to share a significant part of the information. The first activity is to create the mapping, which will contain matches/mismatches or edit operations between a determined target sequence and the reference. After that, tools can either execute the process of Post-processing or Second Order Mapping. 3) Encoding Phase, in this phase the input is the Mapped Sequences that will be encoded, i.e., written in a binary way. The goal is to rewrite instructions aiming to reach the lowest possible entropy. In this phase, some tools opt for grouping data according to the distribution of symbols that will be encoded. This is done because some entropy encoding methods have their efficiency directly related to the number of distinct symbols as well as the probability distribution of each symbol. For example, mismatches can be encoded separately from matches, which can have the element referent to the position, coded separately from elements referring to the size. The final result is a binary file containing one or more compressed sequences.

The 32 assessed vertical genomics compression tools in this review are based on 3 distinct phases (Fig 4), with each one been composed by a different number of steps (Table 6). Such steps are detailed within the next sections.

### Automatic selection or creation of the reference sequence

**Fig 5:**
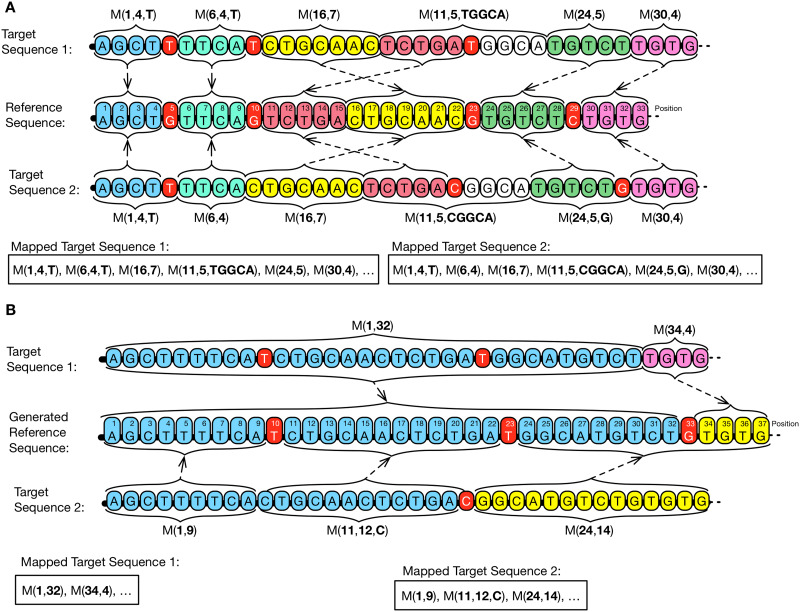
Reference sequence (artificial vs. biological). In this figure we illustrate the differences of using a reference data based on a real genomic sequence (B) compared to an artificially generated sequence (A). The great advantage of using an artificial sequence is its high level of similarity to the to-be-compressed sequences since its creation is based on the most frequent segments found within the collection of to-be-compressed sequences. However, the high computational cost for creation and storing of artificial sequences must be considered before applying this approach.

This step aims to determine at least one sequence that will be used as a reference for the detection of shared segments in the collection of to-be-compressed target sequences. The reference selection can be done by automatic selection from the collection of the to-be-compressed sequences (Fig 5B). Tools implementing this strategy are DNAComp, CoGI, FRESCO, GDC 0.3. Another strategy for determining the reference is based on generating an artificial sequence (a created consensus reference that is based on the to-be-compressed sequences (Fig 5A)) and is implemented by the tools RCC, CoGI, Kanika et al. (44), FRESCO and Project DNA Compression. For both cases, better compression results are reached when higher similarities are found between the reference and to-be-compressed sequences.

According to our analysis, we have identified three distinct forms for reference sequence selection:

- Manual: is manually provided by the user before the start of the compression;
- Automatic: is automatically selected by the compression tool. This process is executed before the compression;
- Generated: is automatically created, where the tool generates an artificial sequence based on the to-be-compressed sequences. This process is executed before or during compression.

Although the selection of a reference has a direct and crucial impact on the final size of the compressed sequences [49, 59, 60], the vast majority of tools require a manual determination, as reported by HiRGC, FM-context, MLF, RLZAP, NRGC, RCSCS, GeCo, SLF, Arram et al., JDNA, ERGC, GDC 2.0, iDoComp, RLZ 1.4, DnaCompact, Dai et al., ABRC, COOL, GRS, GReEn, RLZ-Opt, and RLZ. The efficient selection of reference allows minimizing the amount of variation between reference and target and the total output file size [59]. Therefore, automatic-based reference selection may bring significant benefits in the final compression ratio.

### Reference sequence indexing

Some tools, during the mapping phase (detection of shared segments between genomic sequences—detailed in section 3), use a specific strategy to access the reference sequence content. One of these strategies is based on the use of indexing methods to reduce the computational cost. Indexing-based methods use data structures to speed up the search for stored content. They are mostly based on suffix arrays that allow to locate a segment of size m in a sequence of size n with complexity O(m log n) compared to O(n) without its use. In general, the lower the computational cost to access an index, the higher the cost in its generation and storage [54].

In this context, the tools MLF [61] and SLF [62], use suffix arrays to create two data lists to search for shared segments. One list is the *Longest Previous Factor* (LPF), and the other is the Position (POS). In tests reported by SLF tool, it consumed 2376 seconds to construct the index of the H. sapiens genome (version KOREF_20090131 (41)). For the creation of lists LPF and POS, 399 seconds were necessary, resulting in a total of 2775 seconds. In contrast, the use of these lists reduced the compression time for the largest human chromosomes to less than a second. In the MLF tool, on the other hand, which uses an improved version of the suffix array, it took less time than the SLF, 889 seconds, to create the index of the same genome, but it took about 39 seconds to perform the same sequence compression.

This scenario exemplifies the direct correlation between the time spent for reference-indexing step with the performance of data compression tool. A similar analysis was described by the authors of FRESCO tool, who have identified that, by using the index structure based on a *k-mer* hash table, the memory consumption averaged between 8 to 10 times the size of the reference sequence. With the use of an index structure based on compressed suffix trees, the memory demand was decreased up to 5 times, but with a processing time 30% higher than using a *k-mer* hash table structure. Once again, such observation makes evident that the use of an index structure could substantially boost increase the final performance of the tool.

Based on these performance issues, some tools also implement specific heuristics to get around the rise in computational cost caused by index creation. JDNA tool implements a strategy that creates an on-demand index, that avoids the need to create an index of the whole sequence [see Additional file 1: Section S1]. Instead, JDNA only requires to index an average of only 2, 5% of the sequences, that reduces the required memory for the index. Similarly, other tools such as ABRC and ERGC were based on a strategy that divides the reference sequence into blocks and then starts the indexing process, block by block, and delete it after use.

Among all analyzed approaches, COOL and DnaCompact are the only tools that did not require index creation. To replace the index, they work by limiting the search space to a fixed size window within the reference.

The HiRGC [63] tool presents a new approach in which the mapping process receives *2-bit* integer sequences as input. With this, they do not need to deal with large alphabets. To achieve this, all *non-ATCG* symbols are removed before indexing the reference sequence, and then the sequence is encoded to a *2-bit* integer sequence (*A* = 0, *C* = 1, *G* = 2*andT* = 3). After that, a k-tuple hash table is created based on the values of the tuples—of size k—from the reference sequence. The numerical value of a k-tuple is calculated based on a simple formula created by the authors. A similar process is applied to the target sequence. In the end, all the *non-ATCG* symbols that were removed, are stored along with their respective positions. It is also stored the positions of the lowercase symbols. This information will be used in the decompression process so that the final decompressed file will have precisely the same content as it initially did.

As discussed here, different approaches have been used to deal with reference sequence indexing to attend memory issues. In the assessed tools, a total of 6 types of index structure were identified: *k-mer* hash table, K-tuple hash table, Suffix array [64], FM-index [65], Compressed suffix tree [66], Matrix graph [67] and Longest previous factor [68].

The choice of the type of index structure to be used has a direct impact on processing time and the needed memory space for data processing, and the *k-mer* hash table structure presented the best results compared to the other structures [69]. Other structures for indexing, such as those ones based on FM-index also demonstrated some advantages compared to *k-mer* hash tables, such as: the mapping performance is not influenced by the size of the reference sequence and, given the fact that the reference is rarely altered, it is possible to keep stored the index to compress other target sequences [70]. Despite the importance of an index in the final compression quality, its storage cost demand must be considered, because in some cases, such as in tool Arram et al. [70], the index size generated for the human genome is of 17GB.

Of all assessed compression tools, *k-mer* (or *k-tuple*) hash table is the most frequent type of used structure, being reported by 11 out of 32 tools assessed (HiRGC, NRGC, GeCo, JDNA, ERGC, Kanika et al., GDC 2.0 [50], CoGI, FRESCO [49], GReEn and GDC 0.3). A hash table stores keys and values [71]. When a *k-mer* hash table was used as an index structure, we extracted the *k* value, that corresponds to the segment size in the assessed target sequence (Table 6). Regarding the values of *k*, it is know that the smaller the value, the longer the time needed for index generation and its corresponding size and without any difference in the final compression ratio [49].

Although the high computational cost involved for reference sequence index generation, the analysis of the assessed tools demonstrated that the use of an index can improve the final performance of data compression tools.

### First order mapping

It is a data transformation step responsible for over 70% of the processing time [70]. In data compression, the goal of a transformation phase is to reduce the redundancy of information and the number of unique elements that need to be stored. With that, data disorder is reduced, lowering its entropy, which allows increasing their compressibility at the encoding phase (detailed in section 6). It is through the transformation that it reduces the necessary space for storage in a smaller magnitude than what previewed by Information Theory [46]. This idea was initially explored by LZ transformations [42, 72]. Before that, the encoding phase was fed with the original content of the to-be-compressed data, limiting it to the distribution of each element that composes the input data.

To reach higher efficiency, the transformation step must also be capable of exploring characteristics inherent to the data being processed; in this case, if the genomic sequences considered are resulting from similar organisms, of the same species. In the context of vertical compression of genomic data, the goal is to reduce the number of nucleotide segments that are equal and shared among the to-be-compressed genomic sequences. In the analyzed tools in this review, the following techniques were identified for mapping:

- **Factorization**: The factorization process, based on LZ77 concept [72], tries to pair the current segment with an earlier occurrence of itself. The implementation of this strategy requires a dictionary structure, composed only by words already visited in the input stream. In vertical compression, instead of using, like a dictionary, already visited segments of the input stream, the search for repetitive segments is restricted to other sequences available as references [52]. One particular case is tools RCC, GeCo [47], SLF [62] and DNAComp [73] that include the input stream (the target sequence) in the search space. In this way, besides using only the reference sequence as search space, it uses information from the target sequence being compressed. As a result of this process, matches are generated, which are also known as: factors [52, 74, 75], LZ-matches [69], relative match entry [76], referential match entries [49] or triploid [77]. These matches represent shared segments between sequences and can be interleaved with segments of one or more nucleotides not shared between sequences, known as mismatch bases. Matches can be represented as a) pair (*p*, *l*), where *p* is the starting position of the match at the reference sequence and *l* is the size of the match; or b) triplet (*p*, *l*, *mi*), in which *p* is the starting position of the match, *l* is the size of the match and mi are the mismatch bases. There are several ways of performing factorization and can be based on sliding window concept (COOL and DnaCompact [75, 78]), alignment (ERGC [79]), greedy search (HiRGC [63], FM-context [77], FRESCO and RLZ [49, 52]), look ahead (RLZ-Opt [74]) or fingerprinting (NRGC [55]).
- **Grammar based**: Sequences are processed iteratively so that, at each step, it is attempted to identify frequent segments to be replaced by non-terminal symbols, which are not part of the initial set of symbols in the processing content. The goal is that at each stage, it should be possible to identify longer segments. These segments can also be composed of non-terminal symbols. The process ends when a predetermined number of steps is reached or when sufficient segments are no longer found to be replaced [80]. Among the tools assessed, only COMRAD operates in this way.
- **Alignment**: It is when DNA sequences are aligned to find shared segments. Such alignment can be done by pair, in a way that a target sequence is aligned with a reference sequence, aiming to find the local shared sequence and then extracting the different sequence (implemented by GRS tool [38]). Another search method is to align a set of sequences to extract the consensus sequence that will describe the collection of sequences used (implemented by DNAC-K [81]).

As a result of the first order mapping process, a map is generated that contains information about shared and non-shared segments (Fig 3). The only tools that do not implement such strategy are GeCo [82] and GReEn [39] that do not have a mapping phase. Both are statistical tools, which send the target sequence content directly to encoding.

We have identified that, during mapping, some tools implement specific strategies to execute the search for shared segments, called Local Search. The goal is to increase efficiency in the search for shared segments or in the identification of matches that have a more efficient representation. This more efficient representation will be explored in the encoding phase. This local search, looking for the beginning of the next match, is done by limiting the search space in the reference sequences, so that the search is started in a subsequent position close to the last identified match. Thus, in addition to reducing the need to go through the whole reference sequence whenever we look for a match, it is possible to prioritize match detection so that they are identified in a growing chain. With this, it is possible to create a relative representation of positions. Such behavior is possible given the homology and co-linearity of genomic elements [83] existent between organisms of same species, or between organisms of genetically similar species. With that, the chance that a large segment (a match) is interrupted by mutations, such as SNPs, INDELs, rearranges and CNV is significantly high.

**Fig 6:**
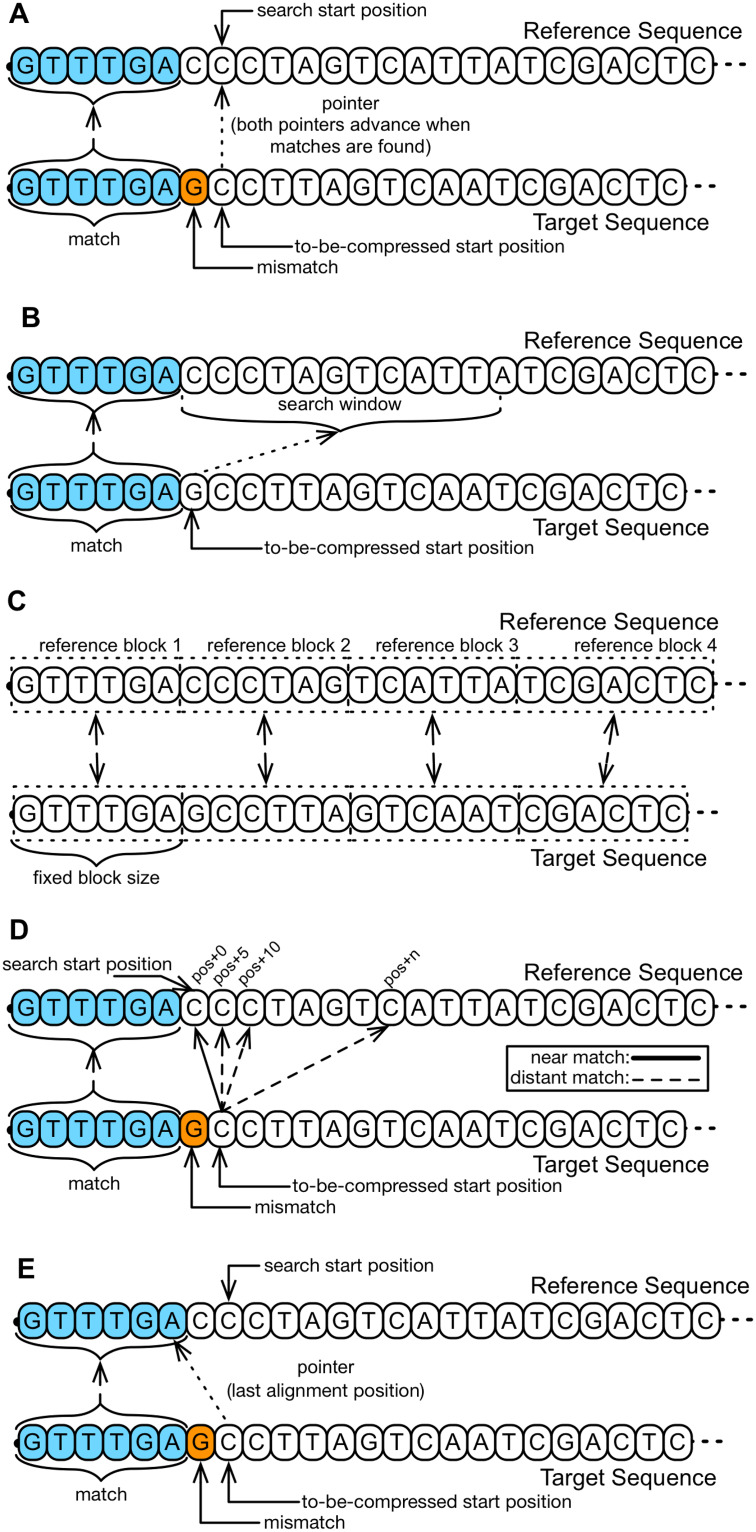
Local search. Illustrative examples of how the local search can be implemented. (A) After identifying a match or a mismatch the pointer advances, at the same time, both in the reference sequence and in the target sequence and then the search is initiated. (B) The search is limited to a search window that advances every match or mismatch. (C) The reference sequence and the target sequence are divided into blocks, and the search for matches is restricted, in a first moment, to such blocks. (D) The search can be made in the whole reference sequence, but penalties are given to possible matches found in distant positions from the last match identified. With that, the tool prioritizes matches closer to the recently identified. (E) The search is always initiated right after the ending of the last match identified.

There are several approaches to the implementation of Local Research behavior, the main ones being highlighted here (Fig 6):

1. Keep a pointer between reference and target (Fig 6A), and advance this pointer at the same time in reference and target for every match or mutation identified, as implemented by tool JDNA [84]. Another possibility, implemented by tool GDC 2.0, is to perform some punctual simple verifications before initiating the search for a larger segment, advancing the pointer in reference [34];
2. Restrict the search within a bidirectional window (Fig 6), which can have its position and size updated dynamically, behavior presented by COOL and DnaCompact tools [75, 78];
3. Splits reference and target in same size blocks and process both blocks into the same order as they appear (Fig 6), giving preference initially to matches in these blocks (presented by tools ABRC and ERGC [76, 79]);
4. Implementing a metric that penalizes matches that are distant from the latest recently considered match (Fig 6), implemented by tool GDC-0.3 [69];
5. Initiate the search for the next large match after the ending of the last recently identified match (Fig 6), implemented by tools GeCo [47], GReEn [39], RLZ-Opt [74], RLZAP [85]. Tools Dai et al. [86] and CoGI [87], however, restrict the search to a nearby region in which recent segments were identified.

The decision to implement Local Search depends directly on the implemented coding scheme. Among the tools evaluated, we identified in 16 of them the Local Research behavior: RLZAP, GeCo, JDNA, ERGC, GDC 2.0, CoGI, DnaCompact, Dai et al., ABRC, FRESCO, COOL, GRS, GReEn, GDC-0.3, RLZ-opt and RLZ-RePair. Some tools are based on close matches identification, as RLZ-opt (74) and GDC-0.3 [69], aiming to reduce the cost for storing the match position. Other tools, such as HiRGC [63], FM-context [77], RLZ [52] and FRESCO [49], suggest that, by considering longer matches, even distant from previous ones, there is a reduction in the final amount of matches that, by consequence, reduces the final storage cost as there are fewer elements that need to be stored. Indeed, FRESCO implements both possibilities. Local search, that looks for short and local matches and a greedy search, that looks for the longest possible matches. It is important to notice that some tools implement more than one Local Search strategy, as RLZ-Opt [74] and JDNA [84], in which it is dynamically determined, during mapping, which local search strategies will be applied. GeCo and GReEn tools, despite not executing a mapping phase, use local search strategy to create and keep their statistical models updated.

Another fundamental problem of vertical compression of genomic sequences is related to the storage of numerical elements referring to the position and size of shared segments [59]. The basic approach is to store the position in absolute value, pointing to the reference position in which the shared segment initiates. However, a genetic sequence may vary the size and can contain from millions to billions of nucleotides. The closer to the ending of this sequence, the larger the numeric values to represent the position. One possible solution would be to store the current position value, based on the difference (delta) from the previous match’s position. Such a strategy is possible when the to-be-compressed genomic sequences are evolutionarily related. Thereby, elements’ disposition, although being interrupted by mutations, tends to be co-linear (83). Thus, the probability that shared segments are encountered in an organized way is high, so that the position in which they occur will create an organized chain. This is exemplified in Fig 3c1, where four matches are presented: M(1,8), M(10,7) and M(17,5). The matches, located in positions 1, 10, and 17, are disposed to create an incremental and linear chain. Approaches to the representation of positions identified are tabulated in Table 6. We have identified a particular case in the FM-context [77], wherein they store the size element as a result of the match size minus a *minlen* (minimum length) parameter. This *minlen* parameter is used to limit the minimum size of the matches during the mapping step. By doing so, they get shorter bit code for the size element.

In the mapping step, tools can, besides searching for exact matches, also search for matches that are reverse complement (COMRAD [80]) or palindromes (DNAComp [73]). Although we performed an in-deep literature review, we only found this behavior in these two tools. However, it is largely known that reverse complement and palindromes are explored by horizontal data compression tools, such as DNAPack [88], Biocompress [25]. Even in DnaCompact, a vertical compression tool, which has a horizontal mode, in the horizontal mode, during the mapping phase, it searches for matches considering reverse complements and palindromes.

Another characteristic identified in some tools was the implementation of a step to identify and treat ambiguous bases. This feature is relevant because the current technologies used for genome sequencing have some computational limitations that make reconstructing the original molecule into a single contig a major challenge [89]. This is mainly due to the presence of regions with high repetitions or regions with low sequencing coverage. It is the case, for example, of the centromere and telomere regions of a chromosome. The result is that bases from these regions are represented, in the final sequence, by the symbol *N*. As it is expected that there will be long segments composed by *N*s, tools HiRGC, RLZAP [85], JDNA, GDC 2.0 and GDC 0.3 [69] implement specific behaviors to deal with that. The identification of ambiguous bases is still necessary even with the recent development of new assembly software and more modern technologies of genetic sequencings, such as Nanopore [90].

### Second order mapping

**Fig 7:**
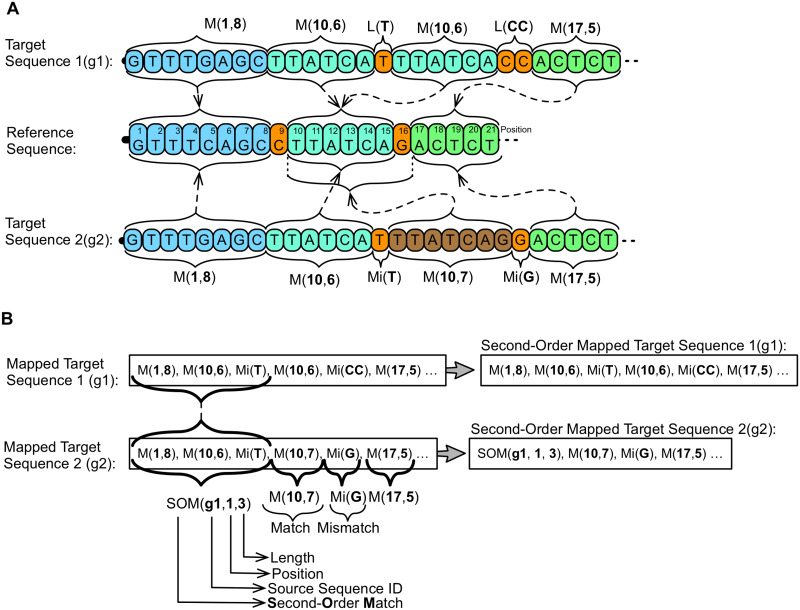
Second-order mapping. In this example, in the first moment (A), two target sequences, Target Sequence 1 and Target Sequence 2, are parsed using a same reference sequence, Reference Sequence. As a result, two mappings are obtained, one for each sequence. In a second moment (B) a Second Order Mapping is executed. The input are the maps previously generated and the tool, parsing Mapped Target Sequence 2 in relation to Mapped Target Sequence 1 identifies a succession of three elements, two matches and one literal that are shared and generate a Second Order Match, SOM(g1, 1, 3). This SOM indicates that, in Target Sequence 2, initiating from position 1 of sequence g1, the first three elements must be considered, in this case M(1, 8), M(10, 6) and L(T).

It is a vertical transformation step that adopts a similar approach to the First Order Mapping step, being identified in tools: GDC 2.0 and FRESCO. Compression ratio gains are obtained because, by compressing DNA sequences of same species organisms, the possibility that the same succession of matches and mismatches occur between distinct target sequences is high [50], and they become proportionally higher as the number of sequences raises [49, 50]. In this way, the search space is composed of the set of already mapped target sequences, and the considered alphabet corresponds to the map of matches/mismatches (Fig 7).

In FRESCO tool, the second order mapping is done splitting the target sequence set into two subsets. The first, that will be the search space for the second order mapping, is composed of the new reference sequences, that will be indexed (hash table). Therefore, the search space has a maximum size. The second set will be composed by the remaining sequences that will be processed, one by one, by checking the search space for successions of coincident matches/mismatches to be replaced by new matches. In the performance evaluations, authors have obtained with Second-Order Mapping, on average, a compression ratio 4 times higher. These results were achieved using a search space of 70 reference sequences.

Tool GDC 2.0 implements a similar strategy. The main difference compared to that implemented by FRESCO is that it increases the search space, including new mapped sequences, to each new target sequence processed by First Order Mapping. Thus, the search space considered by GDC 2.0 tool does not present any size limitations. However, the results reported were based only on the use of Second Order Mapping, which made it difficult to conclusively evaluate the impact of this strategy in improving the compression ratio. In the experiments described, the authors performed tests varying the size of the search space from 10% to 100% of the sequences, obtaining compression ratios ranging from 300:1 to 900:1, respectively. The compression time reduced by 24%, with a decrease of the search space by 50%, while the compression ratio reduced by 26%.

### Post-processing

**Fig 8:**
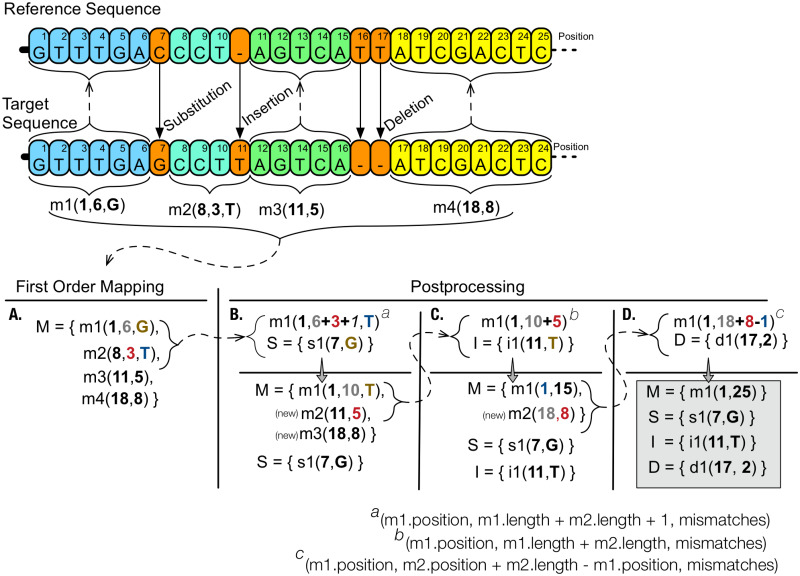
Post-processing. In this example, we explain the post-processing phase. After the first order mapping (A), the set of matches M is processed, two by two, trying to merge them. In the first iteration (B), matches m1 and m2 are evaluated. It is verified that (m1.position + m1.length + m2.length + 1) is equal to m2.position, therefore, there is a substitution between matches. Matches are combined, and m2 is removed from set M, arranging the remaining to such removal. At the same time, instruction s1 is created and inserted in set S that will contain editing instructions of the substitution type. In the second iteration (C), matches m1 and m2 (which was m3) are evaluated. It is verified that (m1.position + l1.position) is equal to m2.position, therefore, there is an insertion between matches. Matches are combined; match m2 is removed from set M, arranging the remaining to such removal. At the same time, instruction i1 is created and inserted in the set I that will contain editing instruction of the insertion type. On the third iteration (D) matches m1 and m2 (which was m4 in the first iteration) are evaluated. It is verified that (2 <= m2.position—(m1.position + m1.length + 1) <= Lmax), where Lmax is a predefined value that determines the maximum size that a deletion can have to be treated by post-processing process. Therefore, there is a 2 bases deletion between the evaluated matches. Once again, both matches are combined, match m2 is removed from set M, which will contain only match m1. At the same time, instruction d1 is created and inserted in set D that will contain editing instructions of deletion type. The goal is to reduce the number of integers. In this case, we initially had 8 distinct integers numbers and at the end, only 6 remained.

This transformation step has a similar goal to Second Order Mapping: to reduce the number of elements to be stored. Tools Arram et al. [70], iDoComp and COOL implement it. The difference in this transformation step, when compared to Second Order Mapping, is in the direction in which data is analyzed: horizontal—where transformations are made on the map of each target sequence using only information contained in the map being processed. Generally, the goal is to merge adjacent matches, separated or not by mismatches. From the analysis of the tools, we identified three different approaches. In Arram et al. [70], it attempts to make a union of adjacent matches that are not interleaved by mismatches. This implementation allowed an improvement of up to 41.3 times in the compression ratio. Tool COOL [75] (Fig 8) evaluates the possibility of matches being separated by insertions, substitutions or small deletions. For each merge, the tool replaces combined matches, creating a larger match and an edit instruction (substitution, insertion or deletion) that is appended to its respective instruction set. In iDoComp [91], in addition to attempting to merge matches that are separate by single-base insertions or substitutions, it tries to merge small matches that are interspersed with small insertions or substitutions that point to distant positions of the previous match. The goal of this second mechanism is to avoid storing small matches that are not a part of a chain known as the *Longest Increasing Sub-Sequence* (LISS). The authors concluded that in these cases it is more efficient to store substitution and insertion instructions than small matches that are not a part of LISS chain. The reason is that these matches imply in need to store large numbers to represent their position, once the delta encoding of these values does not result in small numbers.

### Encoding

This is the only phase implemented by all assessed tools. Such a phase is the last part of the compression process, where the map generated by previous steps is encoded in a binary format. Thus, the goal is to obtain the least possible entropy in the compressed data, in which the smallest amount of bits is sought to represent the most frequent elements of the map.

**Fig 9:**
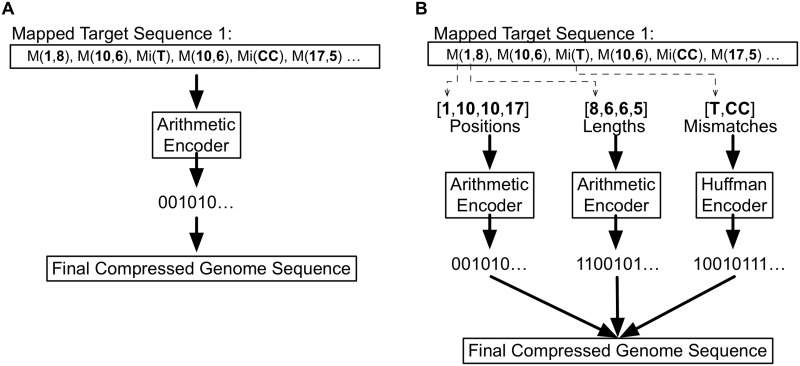
Encoding process. (A) is an example of how a tool performs encoding when all elements that are part of the map are sent to be encoded together. In this case, all elements will be processed in a single data stream. In (B), we illustrate the situation in which a tool splits the elements of the map in three different data streams. The first one containing elements referent to the position of matches; the second one referring to sizes; and, at last, bases that do not compose any match, the mismatches. In this case, each data stream will be encoded separately and, by the ending, the results will be combined into a single file. The advantage of this second approach is that the probability distribution of the elements is more concentrated, which allows for a higher compression rate since, according to Information Theory, smaller binary codes will be conceded to symbols that appear more.

At this stage, each tool can also apply one or more encoding methods for different map elements. This combination is called the coding strategy. For example, GRS [38] applies the same method to the map as a whole, while GDC-0.3 [69] and iDoComp [91] apply different methods to each map element, contextually encoding each data group. In Fig 9 we illustrate both methods described. The first approach (Fig 9A) exemplifies the process in which all elements that compose the map are encoded using the same method. In the second approach (Fig 9B), three data streams are created. The first is applied only to values that indicate positions, the second to values referring to match the size and finally a stream for symbols that are not part of matches, called literals. In this example, each stream is encoded separately and, in the end, the results are combined, generating a single file that corresponds to a compressed representation of the processed sequence. The advantage of this second approach is that each stream has a more homogeneous symbol distribution, which makes it more predictable. Statistical encoders based on the probability distribution of symbols then exploit this homogeneity. Some tools, as JDNA [84] and SLF [62], use other generic compression tools as, for example, GZIP or 7-zip for some elements that compose the mapping.

Considering all papers assessed, we identified the use of 15 encoding methods: Huffman Coding [92], Canonical Huffman Coding [93–95], Golomb Coding [96], Elias Gamma Coding [97], Arithmetic Coding [98], Run Length Coding [96], PPMD [99], which is a variant of Prediction by partial matching (PPM) [100], Range coding [101], GZIP [102], Log-skewed [103], Compressed Integer Set [104], Compressed bit vectors [105], Lempel–Ziv–Markov chain algorithm (LZMA2), Variable length byte coding and Arithmetic Coding with encryption capability [106].

Among all the vertical compression tools assessed, it was also possible to identify that only RCSCS [107] describes the use of methods to ensure protection with data encryption. In this context, the SECRAM [108]—a horizontal genomic data compression tool—is one of the first to consider also the protection based on encryption and access control to compressed genomic data. Another tool, called E2FM [109], that is a self-index for collection of genomic sequences also have this capability, to encrypt the compressed genomic data. However, regarding the fidelity of the information, we did not identify tools with such control.

### Discussion

The demand for storage space of genomic data has become one of the limiting parts on the advance of the genomic era, and data compression is an important alternative to reduce such limitation. In this sense, we investigate, in a broad way, as a form of systematic literature review, all existing tools for vertical compression of assembled genomic sequences stored in FASTA format. In total, 32 tools were identified and characterized in different aspects: the compression scheme and the way they compress a single sequence or a collection of them; the size of alphabets considered; if they use external memory during compression processes; if they allow direct access to compressed data.

From the analyzed works, the execution flow of each tool was obtained, which is constituted by phases of preprocessing, transformation and data encoding. For each phase, specific approaches were identified to deal with: a) selection, indexing and use of reference sequence (Figs 3 and 5); b) search for shared segments between sequences that present specific behavior through the Local Search technique (Fig 6); c) try to reduce the total amount of elements to be stored (Second Order Mapping or Post-processing) (Figs 7 and 8); and d) coding with the use of different techniques for different elements resulting from the mapping phase (Fig 9).

The analysis of the tools also allowed to identify the lack of a standardized methodology to evaluate the performance of vertical genomic data compression tools. Thus, in order to be able to compare different tools qualitatively, a comparative analysis with the same datasets would be necessary. We have also considered that, in order to better evaluate each compression phase, the tools could present some metrics referent to the executed processes. For example, in relation to the transformation phase, details could be described, such as: the total amount of matches between sequences, average match size, total size (in bytes) of matches, amount of mismatches, average mismatches size, total size (in bytes) of mismatches and the final entropy of the generated map. This information could be used to verify which tool (or approach) creates the best map or which one can identify the more significant amount of matches. With this, it would be possible to perform a verification between the percentage of target sequence identified as similar, or the average size of each match related to the final compression ratio. In this way, it will be possible to verify the relationship between the ability to locate similarities concerning the achieved compression ratio. This would also allow evaluating the efficiency of each approach towards different organisms, with different levels of similarity. In the analysis performed, only SLF tool provided the generated map size—this information is crucial to identify how the encoding influenced the final compression ratio.

The results presented by evaluated works demonstrate that the compression tools allow reducing the space needed to store data, regardless of the method considered. One of the main problems in genomic vertical data compression is to find an optimized relation between mappings that captures as many shared segments with the lowest coding costs possible.

### Criteria suggestion for evaluating genome compression tools

As previously mentioned in this systematic review, the data compression process is mostly shared among the proposed tools, which is based on three distinct phases. The phases include: pre-processing, transformation, and coding. Despite this, so that the results presented by each tool can be compared to one another, it is necessary to follow a common evaluation protocol. Such a protocol must contain a minimum set of basic requirements regarding the data being compressed (dataset) and indispensable metrics for the evaluation of the compression performance. However, we did not find in the literature a protocol with a specific objective of standardizing genome compression tool assessment that would fulfill such criteria. To tackle this problem, we propose some rules to be observed so that the results between different genome compression tools can be compared.

**Dataset**: Standardizing the dataset to be used in the compression tests is one of the main points to ensure that the results achieved by compression tools are comparable. We found in the literature two authors who published datasets for genome compression benchmarking [110, 111].

The benchmark dataset proposed by [110] contains 15 genomic sequences. This dataset contains a total of 534,263,017 bases, which is approximately half a gigabyte. Besides, it maintains a consistent balance between the number of strings and their respective sizes. It also reflects the main domains and kingdoms of biological organisms, and thus, it allows a comprehensive and balanced comparison for compression methods. For tools that manipulate FASTA files, it is worthy to highlight that each sequence of this dataset is in raw format, i.e., they do not present headers.

The dataset proposed by [111] consists of sequences from several different organisms, with 1105 prokaryotes, 200 plasmids, 164 viruses, and 65 eukaryotes. Furthermore, the author of this work found a scientific way to select samples for compiling the dataset for the benchmark, using multi-stage sampling strategies. The data in this dataset are composed of FASTA and multi-FASTA files.

Both datasets are well-founded on their representation in the universe of genomic sequences existing in public genomic databases, such as NCBI [112]. However, the first dataset mentioned above introduced in [110] is much smaller, and thus, the processing time is consequently smaller as well.

**Disk/storage usage**: For this point, two metrics are of fundamental importance for the performance evaluation of genome compression tools. These metrics are the space savings, and the number of bits to store a symbol (bits per base, or bpb). The use of these metrics is related to compression using the “Naive bit encoding” [51]. Therefore, the simplest approach to compress a genomic sequence, without the need for any specialized tool, is to assign two bits to each symbol of the genomic alphabet (A, C, G, and T) that represent the nitrogenous bases. As a result, we can verify that, without using advanced techniques, it is possible to achieve a space-saving of 75% using 2-bit coding per base. It occurs because is required 8 bits to store an ASCII symbol in FASTA format. However, when encoding each symbol with 2 bits, there is a 75% space-saving by the equation: *spaceSavings* = (1 − (*compressedSize*/*uncompressed* − *size*)) * 100. Likewise, to calculate the number of bits used to store each symbol, the equation used is the *bpb* = *numBits*/*numSymbols*. The numBits variable represents the number of bits used for data storage after compression, and numSymbols is the number of symbols in the sequence prior to compression. Consequently, it is possible to determine whether the performance of the specialized genome compression tool is better than the 2-bit per base coding baseline.

**Compression/decompression time**: It is relevant to collect this type of information when the compressed file does not allow random access to the data. Thus, whenever it is necessary to access some information on the genome that was compressed, it will be required to decompress the entire genome first. Therefore, the decompression process will occur many times. From this perspective, it is possible to state that the decompression time is more important than the compression time since the compression process will happen once, even though this process takes more time to be completed. Thus, authors can measure the compression and decompression time in seconds using the equation *tc* = (*endTime* − *startTime*) and *td* = (*endTime* − *startTime*), respectively, where tc represents the compression time, td is the decompression time, startTime is the moment when the process is initialized, and the endTime is the moment when the process is finalized.

**Memory and CPU usage**: The discussion of these metrics needs to be deepened in terms of how they are gathered and analyzed. The difficulty lies in knowing the ideal time frame to collect the peak of memory use and the percentage of CPU usage. Depending on the strategy followed, the genome compression/decompression process can use 100% of the available memory and CPU according to the complexity of the algorithms. On the other hand, some tools may use lower memory percentages and reach the maximum memory or CPU usage in only one of the compression/decompression phases. Thus, our suggestion is for the minimum data collection for this metric to capture the peak memory achieved during the compression and decompression process.

**Comparison of performance with baseline tools**: Comparing the compression performance with the results of other specialized genome compression tools as a baseline makes the results more relevant. The relevance is on the possibility to check whether the compression rates of the proposed specialized genome compression tool achieves better results than the compression rates of existing tools.

**Other dimensions discussion**: In addition to the proposed metrics and datasets, there are still other dimensions to be standardized, such as the method of execution of the compression tool, taking into account the order of the arguments, in addition to defining the ideal windows of time for the collection of data from memory and CPU usage. Likewise, it is necessary to standardize also what information should be available to the reader to guarantee the reproducibility of the experiment and how the results will be presented, e.g., using size measures in bytes instead of kilobytes, megabytes, or larger units of this measurement. However, these points need to be deepened on the subject, which is an excellent opportunity for future research and publication of work to propose a protocol for evaluating lossless genomic data compression tools with the vertical approach.

## Materials and methods

The method used for obtaining this SLR was based on the protocol developed by Kitchenham et al. [113]. The main goal of this review was to identify and synthesize the state of the art vis-à-vis the computational tools for the vertical compression of DNA sequences in FASTA format. Such compression must be made with no loss and no use of previous knowledge, for example, a map that contains expected mutations for a determined organism.

The aspects referring to search strategy, inclusion criteria and data extraction strategies will be discussed subsequently.

### Search strategy

The search strategy was structured in two phases. The first one consisted of an exploratory study focusing on the indexes composition, the identification of sources of data and the definition/restriction—in terms of dates—of the investigated period. The second phase consisted of an effort to filtrate, analyze and compare the works recovered by the application of the search strategy. Such phases were fundamental for giving support and systematize the achievement of the final goal in this SLR.

A search string was defined using various keywords and three concepts (Table 3). The selection of such keywords was guided by the main goal and by the research questions. The combination of these keywords originated the following search string: ((compression) AND (genome OR genetic OR dna OR nucleotide OR “biological sequence” OR genomic) AND (relative OR referential OR reference OR model OR dictionary OR codebook OR collection OR multiple OR vertical OR content OR set)). The validation of such search string was done by an iterative process that consisted in creating a previous round of search, and in the confrontation of the result with other reviews and works already known in the literature. During this process, the absence of terms was noticed, such as genomic and content, that were, therefore, included. It was opted not to include any synonym for the keyword compression because based on the results of the initial search—exploratory phase -, no other keyword that was associated with the concept of “data compression” was found.

**Table 3:** Concepts and keywords used to create the search string.

Concept	Keywords
Data compression	compression
Biological data	genome; DNA; genetic; nucleotide; biological sequence; genomic
Vertical compression	relative; referential; reference; model; dictionary; codebook; collection; multiple; vertical; content; set

**Concept**: Concept that was tried to be covered; **Keywords**: Keywords used to attend the respective concept.

Although the main goal of this review orbits around the identification of computational tools of genomic data vertical compression without loss of information, it was opted not to include in the search string keywords that described no loss behavior. Thus, the search string made it possible for the recovery of tools that worked with or without data loss. Such decision was made given the difficulty of identifying keywords that identified the computational tools of genetic data vertical compression without loss and, at the same time, without overly restricting the identification of works of interest.

The data sources considered were two: PubMed and Scopus. Such sources index around 43000 and 35000 periodicals, respectively (Table 4).

**Table 4:** Searched bases for the systematic literature revision.

Base	URL	Indexed content
PubMed^a^	http://ncbi.nlm.nih.gov/pubmed	43000
Scopus^b^	http://scopus.com/	35000

**Base**: Name of the searched base; **URL**: Electronic address of the used search mechanism; **Indexed content**: Approximate amount of indexed journals. PubMed data^a^: http://nlm.nih.gov/bsd/serfile_addedinfo.html; Scopus data^b^: https://www.elsevier.com/solutions/scopus/content.

The research was made using the search interface provided by the data sources, in which the search setting was full text and no data or specific area restrictions. Such restrictions were not considered in order to allow wider amplitude in the search space. This way, the initial set of works to be analyzed was created by the result obtained in the application of the search strategy. The first step was to remove the duplicated papers. This was done automatically, based on papers title, year and authors list. After this, the inclusion criteria were applied to this set in order to determine the set of primary works. These criteria will be now discussed.

### Inclusion Criteria

In order to focus the analysis efforts in texts relevant to the goal of this review, the following inclusion criteria were applied to the initial set of works. Thus, a given work is included in the set of primary works when:

1. It proposes a tool vertical compression of genomic data without loss;
2. It allows to compress a genomic sequence using information from other sequences, this being the definition of vertical compression
3. It allows to manipulate files in the FASTA format;
4. It must not depend on previous information (a priori knowledge) for its operation;
5. It provides an implementation of the proposed algorithm—meaning that works with theoretical proposals were not selected.

The application of inclusion criteria obeyed the following order: first, criteria 1 and 2 were applied on the titles and summaries; and second, criteria 1 to 5 were applied to the complete content of each article.

Among the excluded works, some can be quoted as examples: (a) were based on self-index (E2FM [109], LZ-End [114] and BioFMI [56]) (b) use genomic sequences only as input for performance evaluation, but are not specific to compress biological data (Ray [115] and Cobald [116]); (c) tools of horizontal compression that allow vertical compression, but don’t present algorithm detail (Biocompress [25], Biocompress-2 [51] and Hex-LRE [117]); (d) compress various sequences at the same time without using information in a vertical manner (GtEncseg [118]); (e) don’t work with files in the FASTA format (TGC [119]); (f) need previous information (database with genetic variants already known among same specie organisms) (DNAzip [120] and GenomeZIP [121]) or (g) porting already existing tools to other platforms [122]. The remainder set of works was analyzed more thoroughly, focusing on the data extraction to answer the research questions mentioned previously, and whose results are presented in the following sections.

### Data extraction strategy

The strategy consisted of reading each selected paper and identifying/extracting data, as previously said, to answer the research questions. Such extracted data were structured in data sheets shared in clouds. Three specialist researchers executed this stage independently and the conflicting points were reevaluated and solved individually. Research questions were punctuated based on data extracted from the main text of each work and, when necessary, additional source data were used (website, source-code and project documentation).

In some cases, the authors presented more than one proposal in one work. In these situations, data from all versions were extracted and consolidated. In the results and discussion section, all papers were represented as a single tool. This decision was made since the goal is to trace the current scenario referring to existent works and operationalized approaches. In this context, the need to separate and present each implementation as a distinct tool was not verified.

**Notes on** Table 5

- **a**—Tool implemented in MATLAB using external libraries in C.
- **b**—Not informed.
- **c**—For test reasons, the authors have implemented another version in C++.
- **d**—All nucleotides are convert to lower-case
- **e**—Despite being a multi-referential tool, in the presented results, when compressing large datasets, they have used a single-reference version.
- **f**—Tool only works with more than one reference sequence at Second-order mapping, there being no limit to the maximum number of reference sequences
- **g**—All nucleotides are converted to lower-case and those that are not a ‘atgcn’ symbol are converted to ‘n’.
- **h**—The tool groups the sequences into clusters and, for each cluster, it is created a consensus sequence and it is stored as a representation of each cluster.
- **i**—We are calling this tool by that name because we received the source code from the authors and it was named as RLZ 1.4.
- **j**—Tool only works with more than one reference sequence at Second-order mapping, however, has a limit to the maximum number of reference sequences.
- **k**—All nucleotides are converted to upper-case.
- **l**—The maximum number of reference sequences is limited to 39 sequences.
- **m**—The tool does not allow random access when more than one reference sequence is used.
- **Tool**—Name of tool, or quotation when there is no name.
- **Year**—Publication year of each tool
- **Language**—Main programming language in which each tool was implemented.
- **Scheme**—Compression scheme implemented by each tool.
- **Alphabet**—Largest alphabet (set of distinct symbols) supported by each tool
- **Header**—Denotes if the tools consider or not the FASTA file header in the compression process. When a tool considers the header, then it is restored in decompression to the original value.
- **Memory**—Indicates which tools have specific behavior for external memory (disk) usage during compression/decompression process.
- **Access**—Describes the ways to access the compressed data (without the need of complete decompression).

**Table 5:** Vertical genomic data compression tools.

Tool	Year	Language	Scheme	Alphabet	Header	Memory	Access
RCC	2017	Matlab^a^	RF, CB, SR	*ATCG*	n/i^b^	no	n/i^b^
HiRGC	2017	Java/C++	RF, PB	ASCII distinct	yes	no	n/i
FM-context	2017	C++	RF, PB	ASCII distinct	n/i	no	n/i
MLF	2016	n/i	RF, PB	ASCII distinct	no	no	n/i
RLZAP	2016	C++	RF, CB, SR	*ATCGN*	n/i	no	RA, SR
NRGC	2016	Java	RF, PB	ASCII distinct	yes	yes	n/i
GeCo	2016	C	ST, PB	*ATCG*	no	yes	n/i
RCSCS	2016	Java	RF, PB	n/i	n/i	no	n/i
Arram et al.	2016	FPGA^c^	RF, PB	*ATCG*^d^	no	no	n/i
DNAComp	2015	Matlab^a^	RF, CB, MR^e^	*ATCG*	no	no	n/i
SLF	2015	C	RF, PB	IUPAC distinct	no	no	n/i
JDNA	2015	Java	RF, PB	ATCGN	yes	yes	n/i
ERGC	2015	Java	RF, PB	ASCII distinct	yes	yes	n/i
Kanika et al.	2015	Java	DT, PB	n/i	n/i	no	n/i
GDC 2.0	2015	C++	RF, CB, MR^f^	ASCII distinct	yes	no	SR
CoGI	2015	n/i	RF, CB, SR	*ATCGN*^g^	ignores	no	n/i
iDoComp	2014	C	RF, PB	ASCII distinct	yes	no	n/i
DNAC-K	2014	n/i	RF, CB, MR^h^	IUPAC	n/i	no	n/i
RLZ 1.4^i^	2014	C++	RF, CB, SR	*ATCGN*	n/i	no	RA
FRESCO	2013	C++	RF, CB, MR^j^	*ATCGN*	n/i	no	n/i
DnaCompact	2013	C++	RF, PB	ASCII distinct	yes	no	n/i
Dai et al.	2013	C++	RF, PB	ASCII distinct	n/i	no	n/i
ABRC	2012	C++	RF, PB	ASCII	ignores	yes	n/i
COMRAD	2012	C	DT, CB, MR	IUPAC	n/i	yes	RA
COOL	2012	Python	RF, PB	ASCII^k^	yes	no	n/i
GRS	2011	C/Shell	RF, PB	ASCII distinct	yes	no	n/i
GReEn	2011	C	ST, PB	ASCII distinct	ignores	no	n/i
GDC-0.3	2011	C++	RF, CB, MR^l^	ASCII distinct	yes	yes	RA^m^
RLZ-Opt	2011	C	RF, CB, SR	*ATCGN*	n/i	no	n/i
RLZ RePair	2011	C	RF, CB, SR	*ATCGN*	n/i	no	n/i
RLZ	2010	C	RF, CB, SR	*ATCGN*	n/i	no	RA, SR
P. DNA Comp.	2009	Perl	RF, CB, SR	*ATCG*	ignores	no	n/i

**Scheme**: RF = Referential, SR = Single-reference, MR = Multi-reference, CB = Collection-based, PB = Pair-based, ST = Statistical, DT = Dictionary. **Access** RA = Random Access, SR = Single recovery.

**Notes on** Table 6

- **a**—Despite the tool does not implement an index structure, it uses an external tool called PatterHunter to the search for the shared segments.
- **b**—Uses the generic compression tool 7-zip to compress the final result.
- **c**—Sends each nucleotide from the to-be-compressed sequence directly to the encoder.
- **d**—Sends each nucleotide from the to-be-compressed sequence directly to the encoder.
- **e**—Although it does not have a mapping phase, the tool uses local search whenever there is a new attempt to restart the use of the probabilistic.
- **f**—Not applicable, does not have a position element to be stored.
- **g**—For the first match, the absolute position is stored.
- **h**—Uses variable-length integers to encode position and size elements of matches.
- **i**—Uses the generic compression tool GZIP to compress the final result.
- **j**—By default, uses the shortest sequence as reference and it also has two distinct heuristics for automatic reference selection.
- **k**—Used to represent SNPs position.
- **l**—Used to represent the position element of matches.
- **m**—Not applicable, does not use reference sequence.
- **n**—By default, uses the longest sequence and it also has a specific heuristic for automatic reference selection.
- **o**—Implements a bidirectional search window therefore does not need to create an index on the reference sequence.
- **p**—Uses a generic compression tool PPMDj to compress the final result.
- **q**—Implements a dynamic window based on string matching therefore does not need to create an index on the reference sequence.
- **r**—Uses a modified UNIX diff program.
- **s**—Although it does not have a mapping phase, the tool needs to create an index for the reference sequence because it is necessary to create them.
- **t**—The result of (last match position + last match length + previous factors length) is called ‘expected position’.
- **u**—When this happens, then, it means that the position can be derived based on the last position, so that value is not stored. A bit vector is used.
- **v**—Not informed, does not detail how it works.
- **x**—These techniques were not combined, instead, they were used separately to evaluate the performance of each.
- **Tool name**—Name of tool, or quotation of it when there is no name.
- **Reference sequence selection**—Indicates how reference sequence selection is done or if must be manually informed by the user.
- **Reference sequence indexing**—Presents the method or data structure used by each tool to index the reference sequence. At the same time, for the.
- **Mapping**—Lists which scheme is implemented to map target sequences.
- **Mapping local search**—Denotes which tools implement Local Search strategies.
- **Second order mapping**—Denotes which tools execute a Second Order Mapping phase.
- **Post-processing**.—Denotes which tools execute post-processing phase.
- **Encoding**—Presents which encoding techniques are used in the encoding phase.
- **Relative position representation**—Presents, when applicable, how each tool calculates the values of the matches’ position element for relative.

**Table 6:** Phases executed by each VGDC tool.

TN	RSS	RSI	MP	MLS	SOM	PP	EC	RPR
RCC	G	none^a^	F				Arithmetic coding and adaptive Elias Gamma Coding	(actual matched position—last match position)
HiRGC	M	K-tuple Hash Table k = 20	F				Run Length and PPMD^b^	(actual matched position—last match position)
FM-context	M	FM-index	F				Arithmetic coding and Gamma coding	(absolute position)
MLF	M	Longest Previous Factor	F				AD-HOC Binary Coding lzma2^b^ and PPMD^b^	(absolute position)
RLZAP	M	Matching Statistics (LCP array)	F	X			AD-HOC Binary Coding and Compressed bit-vectors^c^	(actual matched position—last match position)
NRGC	M	k-mer Hash Table 99 k = 11, 12, 13	F				PPMD^b^	(actual matched position—last match position)
GeCo	M	k-mer Hash Table (cache-hash) k = n/a	none^d^	X^e^			Arithmetic coding	n/a^f^
RCSCS	M	n/i^v^	F	X			Integer arithmetic coding (with encryption capability)	n/i^v^
Arram et al.	M	FM-index	F			X	AD-HOC Binary Coding^h^	(absolute position)
DNAComp	A, M	none^a^	F				Arithmetic coding, Elias Gamma Coding and Run-length coding	(actual matched position—target size)^g^
SLF	M	Longest Previous Factor	F				LZMA2^b^	(absolute position)
JDNA	M	k-mer Hash Table k = n/a	F	X			Huffman coding^i^	(actual matched position—last match position)
ERGC	M	k-mer Hash Table k = 21, if fails then k = 9	F	X			PPMD^b^	(actual matched position—last match position)
Kanika et al.	G	k-mer Hash Table k = dynamic size	AT				n/a^v^	n/a^f^
GDC 2.0	M	k-mer Hash Table k = 15	F	X	X		Range coding	(last match position + last match length + previous literals length)—actual matched position
CoGI	A^j^	k-mer hash Table k = n/a	AT	X			Rectangular Partition, AD-HOC Binary (static entropy) and Variable length for the reference sequence	(actual matched position—last match position)
iDoComp	M	Suffix Array	F			X	Arithmetic coding	(actual matched position—last match position) k |actual matched position—(last match position + last match length)| l
RLZ 1.4	M	FM-Index	F				AD-HOC Binary Coding, Compressed bit-vectors^c^ and Run Length coding	(beginning of the actual to-be-compressed position at target—actual matched position at reference)
FRESCO	A^n^, G	k-mer Hash Table k = 34	F	X	X		AD-HOC Binary Coding	actual matched position—(last match position + last match length + 1)
DnaCompact	M	none^o^	F	X			Log-Skewed coding, Elias Gamma coding and Arithmetic coding	(actual matched position—start position of the search window)
Dai et al.	M	none	A(d)	X			Run Length coding, PPMD^p^ and Arithmetic coding	n/i^v^
ABRC	M	Compressed Suffix Tree	F	X			AD-HOC Binary Coding	absolute position together with his respective block number
COMRAD	n/a^m^	none	G				Canonical Huffman coding	n/a^f^
COOL	M	none^q^	F	X		X	Huffman coding and Golomb coding	(actual matched position—last match position)
GRS	M	Matrix Graph^r^	A(v)	X			Huffman coding	(actual matched position—last match position)
GReEn	M	k-mer Hash Table s k = 11	none^d^	X^e^			Arithmetic coding	n/a^f^
GDC 0.3	A, M	k-mer Hash Table *k* = (*alphabet*_*s*_ *ize*^*k*^)< = 4069	F	X			Huffman coding and Variable-length byte coding	beginning of the actual to-be-compressed position at target—actual matched position at reference
RLZ-opt	M	Suffix Array / Matching Statistics (LCP array)	F	X			Golomb coding	if (last match position + last match length + previous factors length) t == actual matched position then the position is discarded else position = actual position—expected position
RLZ- RePair	G	Suffix Array	F	X			Golomb coding	if (last match position + last match length + previous factors length) t == actual matched position then the position is discarded^u^ else position = actual position—expected position
RLZ	M	Suffix Array	F				Compressed integer set	absolute position
P. DNA Comp.	M, G	none	n/i^v^				Huffman, Golomb or Elias Gamma coding^x^	(actual matched position—last match position)

**Selection** (Reference selection): G = Generated, M = Manually, AT = Automatically. **Map**: F = Factorization, A(c,d,v) = Alignment: (consensus, difference, variation) sequence, G = Grammar-based.

**TN** (Tool name); **RSS** (Reference Sequence Selection): G = Generated, M = Manually, AT = Automatically; **RSI** (Reference Sequence Indexing); **MP** (Mapping): F = Factorization, A(c,d,v) = Alignment: (consensus, difference, variation) sequence, G = Grammar-based; **MLS** (Mapping Local Search); **SOM** (Second Order Mapping); **PP** (Post-Processing); **EC** (Encoding); **RPR** (Relative Position Representation).

We thank Bruna Milanese Ávila for the text revision, comments and translation into English. We also thank Gabriel Becchi and Ailton Vieira Pinto Filho from Undergraduate Program in Computer Engineering from PUCPR and Jhonatan Francisco de Paula, for supporting literature search. Also, the authors would like to thank for supporting by *Coordenação de Aperfeiçoamento de Pessoal de Nível Superior* (CAPES).

## Supporting information

S1 Data PRISMA 2009 checklist. (PDF)

S1 Table (XLSX)
